# Levels of serum eosinophil cationic protein are associated with hookworm infection and intensity in endemic communities in Ghana

**DOI:** 10.1371/journal.pone.0222382

**Published:** 2019-09-12

**Authors:** Benjamin Amoani, Bright Adu, Margaret T. Frempong, Tracy Sarkodie-Addo, Samuel Victor Nuvor, Michael D. Wilson, Ben Gyan

**Affiliations:** 1 Department of Biomedical Science, College of Health Sciences, University of Cape Coast, Cape Coast, Ghana; 2 Department of Immunology, Noguchi Memorial Institute for Medical Research, College of Health Sciences, University of Ghana, Legon, Ghana; 3 Molecular Medicine Department, School of Medical Sciences, Kwame Nkrumah University of Science and Technology, Ghana; 4 Department of Microbiology, College of Health Sciences, University of Cape Coast, Cape Coast, Ghana; 5 Parasitology Department, Noguchi Memorial Institute for Medical Research, College of Health Sciences, University of Ghana, Legon, Ghana; Helen Keller International, UNITED KINGDOM

## Abstract

**Background:**

The eosinophil cationic protein (ECP) is a cytotoxic protein mainly secreted by eosinophils granulocytes and plays a role in host defense against parasitic infections. Infection with *Necator americanus* (hookworm) is traditionally diagnosed by the Kato-Katz method which is inherently tedious, subjective and known to underestimate infection intensity. This study aimed to assess levels of serum ECP in relation to hookworm infection intensity.

**Methods:**

Stool samples from 984 (aged 4 to 80 years) participants in a cross-sectional study conducted in the Kintampo North Municipality of Ghana were examined using the Kato-Katz and formol-ether concentration methods. Serum ECP levels were measured by ECP assay kit and compared between 40 individuals infected with hookworm only, 63 with hookworm- *Plasmodium falciparum* co-infection, 59 with *P*. *falciparum* infection and 36 with no infection.

**Results:**

Hookworm infection prevalence was 18.1% (178/984). ECP levels were significantly higher in individuals infected with hookworm only (β = 2.96, 95%CI = 2.69, 3.23, p<0.001) or co-infected with *P*. *falciparum* (β = 3.15, 95%CI = 2.91, 3.39, p<0.001) compared to the negative control. Levels of ECP were similar between those with only *P*. *falciparum* infection and the uninfected control (p>0.05). Increased hookworm intensity was associated with a significant increase in ECP level (β = 4.45, 95%CI = 2.25, 9.11, r_s_ = 0.193, n = 103, p<0.01). ECP threshold of 84.98ng/ml was associated with a positive predictive value (PPV) of 98% (95% CI = 92, 100), and negative predictive value (NPV) of 76% (95% CI = 62, 87) in classifying hookworm infection status with an AUROC of 96.3%.

**Conclusion:**

Serum ECP level may be a good biomarker of hookworm infection and intensity and warrant further investigations to help improve current hookworm diagnosis.

## Introduction

Human hookworm infections are caused by two main parasites, *Ancylostoma duodenale* and *Necator americanus* (*Na*), which along with *Ascaris lumbricoides* and *Trichuris trichiura* make up the main soil transmitted nematodes [1, 2].The adult hookworm resides in the small intestine of the host and feeds on blood from lacerated superficial capillary beds in the gut [3]. Hookworm disease is a leading cause of iron deficiency anemia, malnutrition, and inflammatory enteritis [4–6]. Often overlooked, human hookworm infection is one of the most prevalent chronic infections in the world, affecting approximately 740 million people in developing countries [1, 7], including 156 million children and most of these individuals are found in tropical regions of the world where such infections are linked to poverty [8] and poor sanitation. Ghana is endemic for hookworm, which is focally distributed in many areas of the country, with some communities reporting a prevalence as high as 45% [2], and 59% [9].

The eosinophil cationic protein (ECP) is a highly basic, single-chain potent cytotoxic protein mainly secreted by eosinophil granulocytes and plays a role in host defense against parasitic infections [10]. ECP is released locally, usually in the presence of either a helminth or an allergen [11]. Hence, increased worm burden could lead to increased eosinophilia with subsequent increase in ECP levels in circulation. ECP is detectable in blood, stool, and urine [12–14] and has a half-life of about 45 minutes in circulation [15]. Measured ECP levels can be compared with the egg counts in stool, which is the current WHO gold standard [14]. Although the search for a highly-specific, sensitive, cost-effective, noninvasive diagnostic assay for hookworm is far from over, studies into immune molecules such as ECP may lead a step further in that direction. However, no study has investigated the relationship between hookworm infection and ECP levels in urine, stool or blood. The Kato-Katz technique currently used in assessing hookworm infection is tedious and known to underestimate helminths infection intensity if multiple samples are not examined per patient [14]. A sensitive correlate of hookworm infection may complement current diagnostic tools and help improve patient care. This study assessed serum ECP level in relation to hookworm infection and intensity in endemic communities in Ghana and explored its potential utility as a biomarker for hookworm infection.

## Material and methods

### Ethics statement

The study was approved by the Noguchi Memorial Institute for Medical Research Institutional Review Committee (FWA#: 00001824). All study participants provided written informed consent prior to their recruitment.

### Study site and study design and recruitment of participants

The study was carried out in the Kintampo North Municipality (KNM) located within the forest-savannah transitional ecological zone in the middle belt of Ghana. The area is situated at 8.05° North latitude, 1.72° West longitude and 286 meters elevation above the sea level. The study area covers in total, an area of 7,162 km^2^ with a population of approximately 140,000 in 32,329 households [7]. Community members were predominantly subsistent farmers and rear different farm animals. Such farming activities have been reported to increase the risk of acquiring helminths infections [8]. A durbar was held at each of the study villages during which the purpose and the nature of the study were explained to them in their local dialect. One thousand and sixty-eight (1068) individuals aged from 4–80 years who gave informed consent were interviewed and given containers to provide stool samples. Out of these, 984 individuals provided samples which were used to determine the prevalence of hookworm infection. After the initial baseline sampling, hookworm infected individuals were treated with a single dose albendazole (400 mg).

### Specimen collection and processing

Trained field staff administered a demographic and health questionnaire [2] and provided instruction for the collection of stools in a labeled stool-collection container given to each participant for collection the following day. Fecal samples were collected in a central location and kept cool (25°C) and transported to Kintampo Health Research Center (KHRC) laboratory for microscopic analysis to detect the presence of helminth eggs on the day of collection using the Kato-Katz and formol-ether concentration methods [16]. The intensity of *Na* infections as determined by Kato-Katz method were expressed in eggs per gram (EPG) of feces. Hookworm speciation was carried out for hookworm positive cases by PCR using specific primers [17, 18] and all the samples were shown to be *Na* cases. Finger pricks were made to test for malaria using Rapid Diagnostic Test (RDT) kits (CareStart™ Malaria *Pf*HRP2/pLDH Ag RDT, Access Bio, Inc, USA) in the field. Two drops of whole blood were also used to create thick and thin smears on a microscope slide to confirm the RDT results. The prepared slides were examined by microscopy for the presence of *Plasmodium* species. In addition, *P*. *falciparum*-specific 18S rRNA gene PCR using specific primers, was carried out to detect submicroscopic parasitaemia [17, 19]. All malaria parasitaemia detected were asymptomatic and hence were not referred for treatment.

About 5 ml of venous blood was collected by venipuncture into vacutainer Hemogard SST® tubes (Becton Dickson and Company, Temse, Belgium) for serum separation from participants who tested malaria and hookworm positive and those uninfected as endemic negative controls. Patients co-infected with other helminths *(Hymenolepis nana*, *Taenia solium*, *Trichuris trichiura and Ascaris lumbricoides)* were excluded from the study and treated with a single dose of 400 mg albendazole (Remedica, Limassol, Cyprus). In all 198 individuals provided blood sample for haematological and ECP analysis. About 20 uL of blood were placed in 5 ml Drabkins solution to estimate the eosinophil level using haematology analyser (ABX Pentra 60C+, HORIBA Medical, Rue du Caduce’e, France). The remaining blood sample was separated by centrifugation, and the serum was stored at −80°C for ECP analysis.

### ECP level measurements (ELISA)

Levels of ECP in serum were measured by enzyme-linked immunosorbent assay (ELISA) technique using MESACUP ECP test kit (MBL Co., Ltd., USA) and following the manufacturer’s instructions. Briefly, serum samples were mixed with an assay diluent provided in the kit and then transferred to a 96-well microplate precoated with anti-human ECP antibody. After incubation and washing, 100 μL of horseradish peroxidase conjugated anti-human ECP polyclonal antibody was added and followed with the substrate reagent tetramethylbenzidine/H_2_O_2_. The absorbance of each well was read at 450nm in an ELISA plate reader (BioTek). A computer software (ADAMSEL) was used to convert the optical density values from the ELISA into ECP concentrations. All standards used in the assay were provided in the kit. The assay is based on a polyclonal sandwich-type ELISA with a biotin-avidin-peroxidase amplification step. This ELISA detects human ECP with a minimum detection limit of 0.125 ng/ml and does not cross-react with eosinophil derived neurotoxin.

### Statistical analysis

Data were analysed using R version 3.3. 2 (https://www.R-project.org/) and SPSS Version 24 (Chicago, IL, USA). Proportions such as prevalence were compared between groups (Pearson χ^2^ test). ECP levels were log (base = 10) transformed. Association between ECP levels and infection status was assessed by multiple linear regression adjusting for age and sex with the endemic negative control group as the reference. Linear regression was used to determine the association between ECP level and hookworm eggs per gram (EPG) faeces (infection intensity). The Spearman’s rank correlation was used in determining the correlation between ECP levels and absolute eosinophil count. The Area Under the Receiver Operational Characteristic (AUROC) curve was used to explore the potential utility of ECP as a biomarker for hookworm infection. The 95% confidence interval (CI) of the AUROC was computed with 2000 stratified bootstrap replicates. For all analysis, *P* < 0.05 were considered statistically significant.

## Results

### Demographic and parasitological characteristics of the study population

A total of 984 participants submitted stool samples for parasitological analysis and the hookworm prevalence was 18.1% (178/984). The mean (sd) age of the study participants was 22.8 (17.4) years. There was no significant difference in the geometric mean EPG of stool for individuals infected with only hookworm and those with *P*. *falciparum* and hookworm co-infection. Of those individuals who were infected with only hookworm, 68.1% were classified as having light infection (< 2,000 EPG of feces), 11.6% subjects were moderately infected (2,000–3,999 EPG), four 20.3% were heavily infected (> 4,000 EPG). Similar trend was observed in the individuals with hookworm and malaria parasite co-infection (Table 1)

**Table 1:** Demographic and parasitological characteristics of the study population.

Characteristic	Control	*Na*	*Pf*	*Na/Pf*	P-value
Number	36	40	59	63	
Sex Male (%)	16 (44.4)	17 (42.5)	20 (33.9)	43 (68.3)	0.0013^a^
Female (%)	20 (55.6)	23 (57.5)	39 (66.1)	20 (31.7)	
Geometric mean EPG (95% CI)	-	1182.0 (704.9, 1981.9)	-	1087.5 (782.7, 1511.0)	0.79 ^b^
Intensity of *Na*					
Light infection (%)	0	47 (68. 1)	0	17 (50)	
Moderate infection (%)	0	8 (11.6)	0	7 (20.6)	
Heavy infection (%)	0	14 (20.3)	0	5 (14.7)	

^a^Chi-squared test

^b^t-test. Abbreviations: *Na*, *Necator americanus*; *Pf*, *Plasmodium falciparum; Na/Pf*, *N*. *americanus—P*. *falciparum* co-infection; EPG, egg per gram of stool. The values in parenthesis are percentages (%) and 95% confidence intervals (95% CI)

### Association between parasite infection status and ECP levels

Overall, ECP levels were significantly higher (β = 0.65, 95%CI = 0.48, 0.82, p = 0.015) in male participants than females. In a multiple linear regression analysis adjusting for age and sex with the uninfected group as the reference, ECP levels were significantly higher in subjects infected with *Na* only (β = 2.96, 95%CI = 2.69, 3.23, p<0.001) or co-infected with *Pf* (β = 3.15, 95%CI = 2.91, 3.39, p<0.001) compared to the *Pf* infected group and the uninfected control. However, there was no significant difference in ECP levels between the *Pf* infected group and the uninfected control. Pairwise mean differences of the ECP levels between all the infected groups showed that individuals infected with only *Na* or concurrent *Na/Pf* infection had significantly higher ECP levels compared to those with only *Pf* infection. However, there was no significant difference in the ECP levels between *Na* only and *Na/Pf* co-infected group (Table 2).

**Table 2:** Association between parasite infection status and ECP levels.

Covariate	Log ECP β(95%CI)	P-value
Female	ref	ref
Males	0.65 [0.48, 0.82]	0.015
Uninfected	ref	ref
*Na*	2.96 [2.69, 3.23]	<0.001
*Pf*	0.45[0.20, 0.70]	0.069
*Na* + *Pf* co-infected	3.15 [2.91, 3.39]	<0.001
**Pairwise comparisons of means among only infected groups: Tukey Contrasts**		
*Na—Na* + *Pf*	-0.29 (-0.91, 0.32)	0.605
*Pf–Na* + *Pf*	-2.75 (-3.30, -2.19)	<0.001
*Pf–Na*	-2.46 (-3.08, -1.83)	<0.001

Multivariate multiple linear regression analysis adjusting for age and sex. *β*: Estimated effect of covariate on ECP level, CI: Confidence interval, *Na*, *Necator americanus*; *Pf*, *Plasmodium falciparum*. Multiple R-squared: 0.63.

### Geometric mean levels of ECP and eosinophil count among hookworm positive and hookworm negative participants

**Fig 1:**
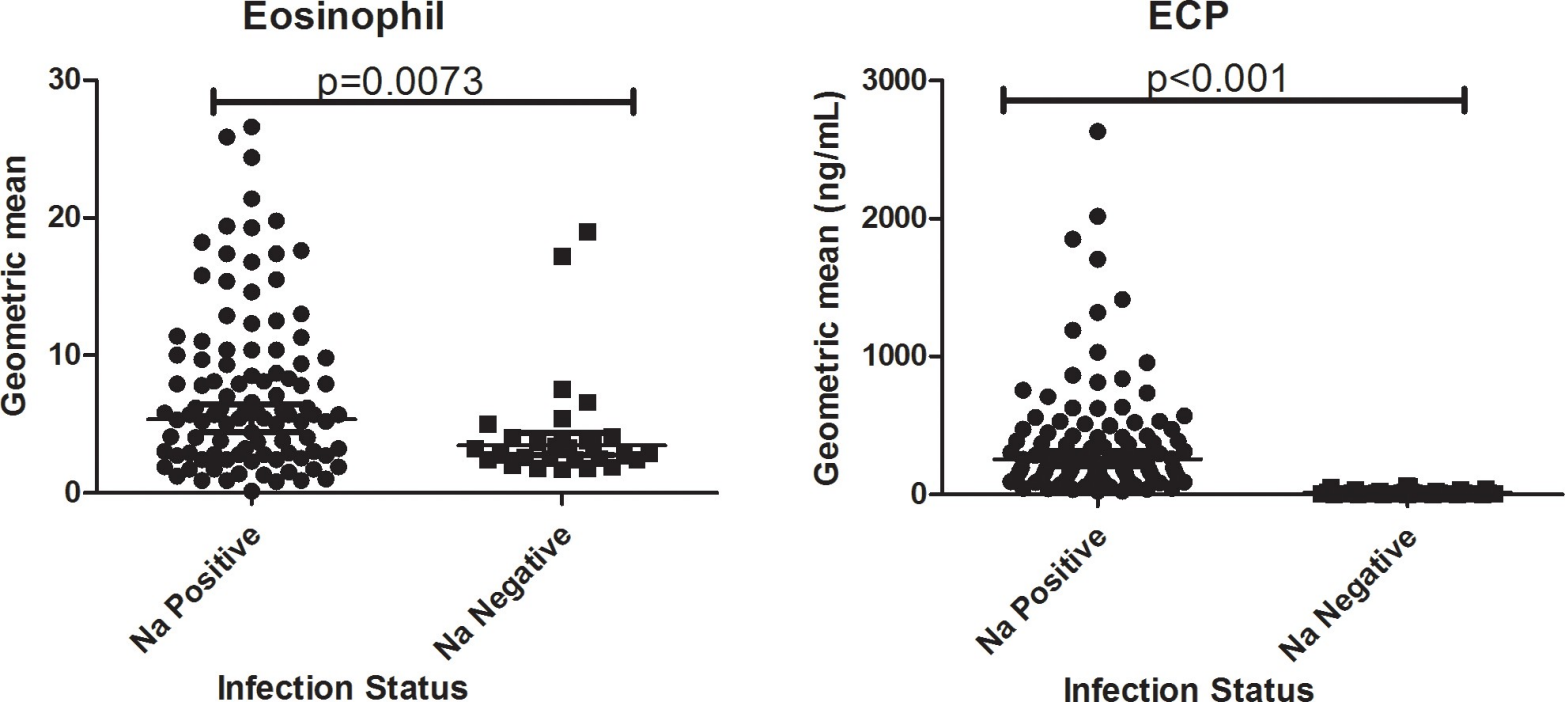
Geometric mean level of eosinophil, and ECP among hookworm positive (103) and negative participants (36). ECP; Eosinophil cationic protein, Na; *Necator americanus*. Error bars are 95% confidence interval (CI).

The geometric mean serum levels of ECP, and eosinophil count were significantly higher in the hookworm positive participants than the hookworm negative participants (p<0.05) (Fig 1)

### Association between *N*. *americanus* infection intensity and ECP levels and relative eosinophil count

The relationship between ECP levels and the intensity of *Na* infection (EPG) was assessed by linear regression model, adjusting for age and sex. Increased in *N*. *americanus* intensity (EPG) led to a significant increase in ECP level (β = 4.45, 95%CI = 2.25, 9.11, r_s_ = 0.193, n = 103, p<0.01) among hookworm infected participant. There was a positive correlation between ECP level and relative eosinophil counts (Spearman’s *r* = 0.382; *P*<0.001). The linear regression and the correlation analysis were done for only those participants with hookworm infection (*Na* only = 40, *Na/Pf* co-infection = 63)

### Predicting hookworm infection with ECP level

**Fig 2:**
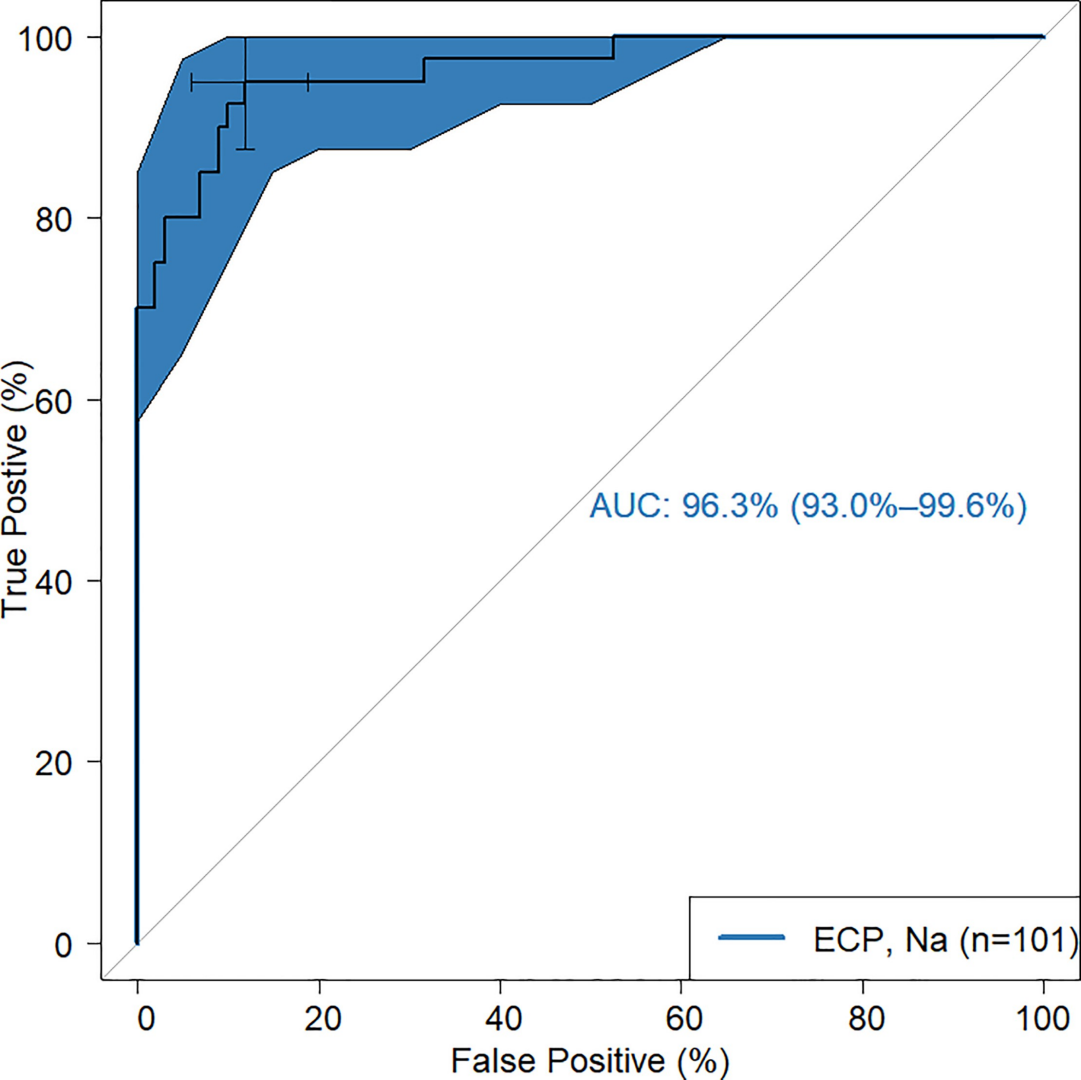
Predicting hookworm infection with ECP level. AUROC curve analysis using ECP levels as a classifier of hookworm infection status was used to predict hookworm infection. 95% Confidence Interval was computed with 2000 stratified bootstrap replicates. AUCROC; area under the receiver operating characteristic curve, ECP; eosinophil cationic protein, *Na*; hookworm.

Area under the Receiver Operating Characteristics (AUCROC) curve analysis using ECP levels as a classifier of hookworm infection status was used to predict hookworm infection. ECP threshold of 84.98ng/ml was associated with a positive predictive value (PPV) of 0.98 (95% CI = 0.92, 1.00), and negative predictive value (NPV) of 0.76 (95% CI = 0.62, 0.87) in classifying hookworm infection status with an area under the curve (AUROC) of 96.3%. Nevertheless, the number needed to diagnose (NND) a person as hookworm positive is 1.20 (95% CI = 1.07, 1.58). In other words, about 12 persons need to be tested to return 10 positive tests. The 95% CI was computed with 2000 stratified bootstrap replicates (Fig 2).

## Discussion

The study aimed to assess levels of serum ECP during hookworm infections and to explore its potential utility as a biomarker for hookworm infection. Serum ECP levels were compared among individuals infected with hookworm (*N*. *americanus*) only, *P*. *falciparum* only, hookworm- *P*. *falciparum* co-infection and negative endemic control. The ECP levels were elevated in hookworm infection but not in malaria infection. This result is similar to finding by Tischendorf et al. [20], who showed that ECP levels were elevated only in diseases that are associated with eosinophilia. The observed higher levels of ECP in hookworm infected individuals is consistent with other studies [14, 20, 21], which reported higher levels of ECP in helminthic infection. Furthermore, we observed that the ECP level among individuals with hookworm infection alone was not significantly different from that of co-infected individuals. Thus, it is likely that the higher ECP level observed in the co-infected individual was mainly due to hookworm and not the presence of *P*. *falciparum* infection. This notion is further supported by the observation here that ECP levels were similar when compared between subjects infected with *P*. *falciparum* alone and those with no infections. However, other parasitic infections as well as allergies have been associated with elevated plasma ECP levels [21–23] and should be accounted for in studies investigating its association with any specific infection.

It is not clear why males had higher ECP levels than females although similar findings have also been reported in a previous study of *S*. *haematobium* infection [14]. Here, it may be due to the higher prevalence (68%) of hookworm-*P*. *falciparum* coinfection among males than females since ECP levels were highest among the coinfected group. The observed positive correlation between relative eosinophil count and ECP level agrees with previous studies [22, 23], while others [20] found no such association. Immune responses to hookworm larvae involve a series of cellular and molecular interactions with immunoglobulin E-activated mast cells [24]. This results in the release of several chemical mediators that attract increased numbers of eosinophils and other leukocytes to the site of infection to induce the release of inflammatory molecules such as ECP, leading to the killing or expulsion of the invading helminth from the body [12, 25]. This may account for the observed higher levels of ECP and relative eosinophil count in the hookworm infected individuals than the hookworm uninfected individuals in our study.

Serum ECP levels increased with increasing hookworm infection intensity (EPG) as has been reported for other helminthic infections [14, 21]. Thus, ECP may be a good predictor of helminth infection intensity in general and further investigation to establish potential specific thresholds for different helminths would be essential. In the current study, infections with other helminths apart from hookworm were low in the study population and were excluded from the analysis.

Serum ECP levels was a strong discriminator of hookworm infection from those without hookworm in the AUROC curve analysis suggesting it hold potential as a hookworm diagnostic marker. Individuals with serum ECP level of at least 84.98 ng/ml were likely to be tested positive to hookworm infection by microscopy 98% of the time and this threshold would classify microscopy negative individuals as negative 76% of the time. However, since ECP is not only involved in defense against hookworm infection but other helminths and immune processes [14, 21], it would be important to compare these findings with other helminth infections as well. This will be vital in the development of any ECP based hookworm-specific diagnostics. Unfortunately, the numbers of such infections in this study were too low for such investigations. Notwithstanding, the current study suggest that, at least serum ECP levels may be useful in identifying individuals with hookworm infections. For a disease mostly endemic in poor tropical regions, any hookworm diagnostic test must not only be sensitive but also highly affordable and easily implemented in such resource limited areas. An ELISA-based test would be too expensive and impractical for remote villages without elaborate laboratory set up but where this test may be needed most. It is therefore envisioned that an ECP-based hookworm diagnostic tool would be developed as a rapid diagnostic test (RDT) kit like what is currently used for malaria and other common infections in order to make it both affordable and easy to deploy in endemic areas. The estimated average cost to develop ECP-based hookworm RDT kit could be about US$1 to US$2.5.

In conclusion, this study shows that serum ECP levels were significantly associated with hookworm infection and intensity and further investigations to assess its development as a hookworm diagnostic tool are warranted.

## Supporting information

S1 Table The demographic, parasitological and ECP data obtained from the study participants. (XLSX)
